# Cyclooxygenase-2 Modulates Glycosaminoglycan Production in the Skin During Salt Overload

**DOI:** 10.3389/fphys.2020.561722

**Published:** 2020-10-23

**Authors:** Róbert Agócs, Domonkos Pap, Dániel Sugár, Gábor Tóth, Lilla Turiák, Zoltán Veréb, Lajos Kemény, Tivadar Tulassay, Ádám Vannay, Attila J. Szabó

**Affiliations:** ^1^ 1st Department of Pediatrics, Semmelweis University, Budapest, Hungary; ^2^ MTA-SE (Hungarian Academy of Sciences - Semmelweis University) Pediatrics and Nephrology Research Group, Hungarian Academy of Sciences and Semmelweis University, Budapest, Hungary; ^3^ MS (Mass Spectrometry) Proteomics Research Group, Research Centre for Natural Sciences, Budapest, Hungary; ^4^ Department of Inorganic and Analytical Chemistry, Budapest University of Technology and Economics, Budapest, Hungary; ^5^ Department of Dermatology and Allergology, University of Szeged, Szeged, Hungary; ^6^ MTA-SZTE (Hungarian Academy of Sciences – University of Szeged) Dermatological Research Group, University of Szeged, Szeged, Hungary; ^7^ HCEMM-USZ (Hungarian Centre of Excellence for Molecular Medicine - University of Szeged) Skin Research Group, Szeged, Hungary

**Keywords:** sodium storage, hypertonicity, dermal fibroblast, skin, cyclooxygenase-2, prostaglandin E2, glycosaminoglycan, hypertension

## Abstract

Sodium (Na^+^) can accumulate in the skin tissue, sequestered by negatively charged glycosaminoglycans (GAGs). During dietary salt overload, the amount and charge density of dermal GAG molecules – e.g., hyaluronic acid (HA) and chondroitin sulfate (CS) – increases; however, the regulation of the process is unknown. Previously, it has been demonstrated that the level of cyclooxygenase-2 (COX-2) activity and the content of prostaglandin E2 (PGE2) are elevated in the skin due to high-salt consumption. A link between the COX-2/PGE2 system and GAG synthesis was also suggested. We hypothesized that in dermal fibroblasts (DFs) high-sodium concentration activates the COX-2/PGE2 pathway and also that PGE2 increases the production of HA. Our further aim was to demonstrate that the elevation of the GAG content is ceased by COX-2 inhibition in a salt overloaded animal model. For this, we investigated the messenger RNA (mRNA) expression of COX-2 and HA synthase 2 enzymes as well as the PGE2 and HA production of DFs by real-time reverse transcription PCR (qRT-PCR) and ELISA, respectively. The results showed that both high-sodium concentration and PGE2 treatment increases HA content of the media. Sodium excess activates the COX-2/PGE2 pathway in DFs, and COX-2 inhibition decreases the synthesis of HA. In the animal experiment, the HA- and CS disaccharide content in the skin of male Wistar rats was measured using high performance liquid chromatography-mass spectrometry (HPLC-MS). In the skin of rats receiving high-salt diet, the content of both HA‐ and monosulfated-CS disaccharides increased, whereas COX-2 inhibition blocked this overproduction. In conclusion, high-salt environment could induce GAG production of DFs in a COX-2/PGE2-dependent manner. Moreover, the COX-2 inhibition resulted in a decreased skin GAG content of the salt overloaded rats. These data revealed a new DF-mediated regulation of GAG synthesis in the skin during salt overload.

## Introduction

While the detrimental effect of sodium (Na^+^) on hypertension is still being discussed heatedly, a new concept of regulated Na^+^ storage in the skin has emerged. Animal experiments verified the role of skin as a Na^+^ depository, which was confirmed by high resolution ^23^Na-MRI imaging in humans (Ivanova et al., 1978; Titze et al., 2003; Kopp et al., 2012; Linz et al., 2015). According to certain research data, Na^+^ concentration in the skin following salt consumption may exceed the level of 140 mmol/L (Wiig et al., 2013). In the model proposed by Machnik et al. (2009), the glycosaminoglycan (GAG)-bound Na^+^ ions generate local hypertonicity. This hypertonicity is sensed by the large number of macrophages simultaneously infiltrating the skin interstitium. Utilizing the cyclooxygenase (COX)-2-VEGF-C signaling pathway macrophages facilitate lymph capillary formation ultimately resulting in the clearance of the excess Na^+^ which has previously been deposited in the interstitium (Machnik et al., 2009; Müller et al., 2013; Zhang et al., 2015).

A large fraction of GAG molecules is found in the skin. GAGs are high molecular weight, long-chained, polyanionic, polysaccharide molecules in the extracellular matrix (ECM), and on the surface of cells. GAGs can either be unsulfated [hyaluronic acid (HA)], or sulfated [chondroitin sulfate (CS), dermatan sulfate, heparan sulfate, and keratan sulfate] in distinct positions. Due to their high charge density, they are capable of binding positively charged ions, including Na^+^, an ion present abundantly in the extracellular space (Farber et al., 1957).

The amount of skin Na^+^-binding GAGs positively correlates with skin Na^+^ content (Fischereder et al., 2017; Sugar et al., 2018). High salt consumption increases Na^+^ content as well as the amount of GAGs of the skin (Titze et al., 2003, 2004), while in salt depletion, both of them decrease (Schafflhuber et al., 2007; Sugar et al., 2018; Lopes-Menezes et al., 2019). Consensus holds that the Na^+^ storage mediated by GAGs is an actively regulated process. GAGs respond to altered Na^+^ concentration of the interstitium by changing their amount, and their charge density. Serving as a buffer against Na^+^ overload, Na^+^ storage on GAGs may blunt blood pressure (BP) rise (Titze et al., 2002; Olde Engberink et al., 2019). This phenomenon implies a strict quantitative and qualitative regulation of skin GAGs; however, the underlying mechanisms to date are not known in detail.

Prostaglandins are paracrine/autocrine regulators, with an important role in the regulation of Na^+^ balance and BP. COX enzymes are the rate-limiting factors in the synthesis of prostaglandin mediators. In the case of salt overload, both the expression of the inducible COX-2 isoform and the amount of prostaglandin E2 (PGE2) rise in the kidney medulla (Yang et al., 1998; Ye et al., 2006; Chen et al., 2008). PGE2 in turn hampers Na^+^ reabsorption in the thick ascending limb of loop of Henle and in the collecting duct – resulting in increased Na^+^ – and water excretion (Ando and Asano, 1995; Hebert et al., 1995; Guan et al., 1998).

COX-2 inhibitors slow down the formation of skin lymph capillaries and cause Na^+^ retention in the kidney (Harirforoosh and Jamali, 2005; Zhang et al., 2015; Yang and Liu, 2017). These consequences (impaired lymph capillary density in the skin and renal Na^+^ retention) lead to salt-sensitive hypertension (Ye et al., 2006; Zhang et al., 2015), a well-known side effect of COX-2 inhibitors (Johnson et al., 1994; Muscara et al., 2000; Hocherl et al., 2002).

Dermal COX-2/PGE2 system is affected by the consumption of a high-sodium diet, too. In a salt overload condition, expression of COX-2 is induced in macrophages resulting in enhanced levels of PGE2 in the skin (Zhang et al., 2015). Keratinocytes have been identified in this process; also, they exhibited both elevated COX-2 activity and PGE2 production when exposed to a high Na^+^ environment (Xu et al., 2015; Zhang et al., 2015). However, other cells i.e., fibroblasts may also be involved since they express COX-2 enzyme, too (Shiraishi et al., 2008; Schirmer et al., 2010; Li et al., 2015; Saalbach et al., 2015).

Experimental data imply that PGE2 strongly induces HA synthesis *via* the cAMP-dependent signaling pathway in fibroblasts and pericardial mesothelial cells by increased HAS1 and HAS2 expressions (Yaron et al., 1978; Honda et al., 1993). PGE2 is also a potent activator of dermal fibroblasts (DFs) during wound healing (Xu et al., 2015). In several other tissue types, evidence has been found suggesting the interconnectivity of the COX-2 system and the GAG metabolism (Schmitz et al., 2003; Rugheimer et al., 2008).

Therefore, we hypothesized that the COX-2/PGE2 pathway may be the most likely element in the regulation and/or synthesis of skin GAGs after salt overload. Our primary goal was to examine the effect of a high Na^+^ environment on COX-2 activity and HA synthesis in cultured primary human dermal fibroblasts, i.e., – the cell type responsible for the production of the bulk of ECM components. We also studied the conceivable interconnectivity between the two regulatory systems. Our further aim was to examine the effect of the COX-2 inhibitor on the Na^+^, CS, and HA content in the skin of salt overloaded rats.

## Materials and Methods

### Ethical Approval

All animal procedures were approved by the Committee of the Care and Use of Laboratory Animals in the Council of Animal Care at Semmelweis University, Budapest, Hungary (PEI/001/1731-9/2015). Isolation and culture on human dermal fibroblasts was approved by the local Ethics Committee of the University of Szeged, Hungary (CSR/039/00346-5/2015).

### Confirmation of Compliance

The investigators understand the ethical principles under which the journal operates and their work complies with this animal ethics checklist.

### Animals

Healthy, 8-week-old, drug‐ and test-naive, male Wistar rats (RRID:RGD 13508588; Toxi-Coop Toxicological Research Centre, Dunakeszi, Hungary) were housed in an accredited, non-SPF animal care facility in a temperature-controlled room (22 ± 1.0°C) with constant light/dark cycles (12/12 h). The initial weight of the animals was 288 ± 9 g.

### Experimental Design and Groups

Two types of dietary regimes were applied: low-salt diet (LSD) consisting of extremely low-salt rat chow (<0.1% NaCl, a virtually salt-deficient diet; Ssniff Gmbh, Soest, Germany) and tap water and high-salt diet (HSD) consisting of Na^+^ rich rat chow (8.0% NaCl; Ssniff Gmbh) and isotonic saline as drinking water. Rats had *ad libitum* access to food and drink. According to our breeding program, the food was exchanged every 2 days. Rats were treated daily with isotonic saline as a vehicle (V) or with celecoxib [C; 20 mg (kg body wt)^−1^ day^−1^; Celebrex, Pfizer, NY, United States] administered by oral gavage. Two hundred micrograms celecoxib was dissolved in 5 ml saline, and the appropriate volume of the solution was administered according to body weight.

Following 1 week of acclimatization, the rats were randomized into three groups (*n* = 8/group): (i) LSD + V; (ii) HSD + V; and (iii) HSD + C and were treated for 2 weeks. The experimental unit was a cage of animals (two rats/cage). The number of independent replications of this experiment was 1 (*n* = 1). The sample was calculated by power analysis. Type I error (α) and the power of study values were 0.05 and 0.8, respectively.

### Arterial Blood Pressure

Systolic and diastolic BP were measured non-invasively on the tail vein of conscious animals using a CODA tail cuff standard monitoring system (Kent Scientific Corporation, Torrington, United States) once every week. Measurements were performed at the same time of the day, from morning to noon. This device uses a clinically validated, proprietary volume pressure recording technology (Kurtz et al., 2005). Several measurements during the acclimatization period allowed the animals to get used to the stress exerted by the measurement.

### Tissue Harvest

At the end of the experiment, the animals were anesthetized by using an intraperitoneal ketamine-xylazine injection: 75 mg (kg body wt)^−1^ ketamine (Richter Gedeon, Budapest, Hungary) and 10 mg (kg body wt)^−1^ xylazine (Medicus Partner, Biatorbagy, Hungary). Blood samples were taken from the abdominal aorta at the end of the experiment. Urine samples were obtained by bladder puncture. The carcasses were skinned, and the skin samples were stored at −80°C until further investigation.

### Determination of Serum and Urine Electrolytes

Na^+^ and creatinine concentration of blood and urine samples were analyzed with a Beckman Coulter AU480 Chemistry System (Beckman Coulter, CA, United States).

### Determination of Skin Na^+^ Content

Skin samples were desiccated at 90°C for 72 h, and then were frozen in liquid nitrogen and pulverized mechanically. Thereafter, 1 g of the desiccated skin samples was dissolved in 5 ml HNO_3_ overnight followed by adding 3 ml H_2_O_2_ (30%) to it. Samples were digested using microwave heated teflon bombs. Homogenized samples were diluted to a final volume of 25 ml and filtered through a 0.45 μm pore size syringe filter. Na^+^ concentration in the solutions was measured with a PFP7 type flame photometer (Buck Scientific, Norwalk, United States). In order to eliminate eventual disturbances which ionization may cause, separate series of dilution were made to calibrate Na^+^ content. Every time, the standard of the other alkali metal was added to the standard series of dilution in a concentration as the sample to be measured required (Dipietro et al., 1988; Chen et al., 2005).

### CS Extraction From Skin and Chondroitinase ABC Digestion

Fifty milliliter lysis buffer consisting of 4% SDS in 100 mM Tris pH 8.0 was added to dry skin tissue. Samples were thoroughly vortexed, and lysis was performed by heating the samples at 97°C for 30 min. The samples were then centrifuged at 16,000 *g* for 10 min, and 2 μl media was taken out from each sample for protein concentration determination. The media samples corresponding to 200 μg protein content were pipetted-off and were transferred to clean Eppendorf tubes, and 9× volume ice cold ethanol was added to precipitate the proteins overnight at −20°C. Next day, the samples were centrifuged at 4°C, 14,000 *g* for 20 min, and the pellets were washed twice by ice cold ethanol to remove the detergent. Then, the pellets were air dried and re-dissolved in 8 M urea, 50 mM ammonium bicarbonate and 5 mM DTT solution at a concentration of 5 μg/μl and incubated at 37°C for 30 min. Samples were alkylated in the presence of 10 mM iodoacetamide at room temperature in the dark for 30 min. The samples were then diluted 10-fold by 50 mM ammonium bicarbonate and trypsin (Promega, Madison, WI, United States) was added at a ratio of 1:50 ratio and incubated at 37°C overnight. Next day, 0.5 μl formic acid was added to quench digestion, and the samples were applied on 10 kDa centrifugal membranes and centrifuged at 14,000 *g* for 15 min. The samples were washed twice with 50 μl 50 mM ammonium bicarbonate and centrifuged at 14,000 *g* for 8 min each time. The GAG chains were retained on the membrane and digested with chondroitinase ABC (Sigma, St. Louis, MO, United States) enzyme solution for 6 h at 37°C. The enzyme solutions consisted of 36.5 μl water, 45 μl 100 mM Tris pH 8.0 buffer, 4.5 μl 100 mM ammonium acetate, and 4 μl 5 mU/μl chondroitinase ABC. Following the 6-h incubation process, 50 μl 100 mM ammonium bicarbonate was added to each sample, and the digestion products were centrifuged at 14,000 *g* for 10 min. The samples were washed twice with 50 μl 50 mM ammonium bicarbonate and centrifuged at 14,000 *g* for 8 min each time.

### Liquid Chromatography-Mass Spectrometry

Prior to the HPLC-MS analysis, CS digests were cleaned using TopTip C18-graphite spintips. The SPE spintip was conditioned with 200 μl 80% ACN 0.1% TFA, then washed with 300 μlwater. The samples were applied in 50 μl water and reapplied once. The salts were washed with 150 μl water, finally, the CS disaccharides were eluted with 150 μl 40% ACN 0.05% TFA. The eluate was dried down and dissolved in 15 μl 10 mM ammonium formate in 75:25 v/v ACN:water (pH 4.4) and 1 μl portions were injected without trapping. For microscale HPLC-MS investigation, a recently published method was used, as follows (Salt gradient chromatographic separation of CS disaccharides, Journal of Chromatography A, under review). An in-house packed HILIC-WAX capillary column (250 μm i.d.) was mounted on a Waters® nanoAcquity UPLC system (Waters, Milford, MA, United States) coupled to a high-resolution Waters® QTOF Premier™ Mass Spectrometer (Waters, Milford, MA, United States) *via* a normal electrospray ionization source. The column temperature was adjusted to 45°C by using an AgileSleeve capillary heater with MonoSleeve column heater controller (Analytical Sales and Services Inc., Flanders, NJ, United States). The flow rate was set to 8 μl/min, as described before (Toth et al., 2019). Eluent A was 10 mM ammonium formate in 75:25 v/v ACN:water (pH 4.4); Eluent B was 65 mM ammonium formate in 75:25 v/v ACN:water (pH 4.4). The gradient program was the following: starting from 6% B, the eluent ratio changed in 0.5 min to 12% B, and then in 4.5 min to 60% B. As a washing step, the composition was elevated to 100% B and held for 4 min, and it was followed by a 5-min-long equilibration at the starting condition.

The mass spectrometry parameters were optimized for the highest sensitivity avoiding undesirable fragmentation in the ion source (Toth et al., 2019). The capillary voltage was set to 2.4 kV, sampling cone to 20 eV, extraction cone to 4 V, and the ion guide to 1.5. The source temperature was 80°C, the desolvation temperature was 100°C, the cone gas was 25 L/h, and the desolvation gas 300 L/h. The investigated compounds were measured as singly-charged anions [deprotonated molecules, (M-H)^−^]. Multiply charged ions or adduct forms complicating the analysis were not observed.

Peaks were integrated with the QuanLynx add-in of Waters MassLynx 4.1 software, and then manually validated.

Relative content of skin HA disaccharides was measured by HPLC-MS as previously described (Sugar et al., 2018).

### Isolation of Human Dermal Fibroblasts

Primary human dermal fibroblasts were isolated from a healthy individual undergoing plastic surgery as described earlier (Kemeny et al., 2016). Fibroblasts used for experiments were from the same individual.

### Cell Cultures and Treatment Groups

Primary, human, and dermal fibroblasts were cultured in Dulbecco’s Modified Eagle’s Medium (DMEM; Gibco, supplied by Thermo Fisher Scientific, Cat. No. 41965062, Carlsbad, CA, United States) supplemented with a 10% fetal bovine serum (FBS; Gibco, Cat. No. 10500064), 1% L-glutamine and 1% penicillin/streptomycin, incubated in a humidified incubator with 5% CO_2_ at 37°C. The cells were plated on six-well plates (5 × 10^5^ cells/well; Sarstedt, Nümbrecht, Germany) without FBS supplementation for 24 h to reach cell adherence. Then, the medium was changed, and the cells were subjected to normal (NS; 150 mmol/L) or high Na^+^ concentration (HS; 200 mmol/L; Sigma-Aldrich, Cat. No. S6191, Darmstadt, Germany) with or without additional treatment of 40 nM PGE2 (Santa Cruz Biotechnology, Cat. No. sc-201225, Dallas, TX, United States) or 4 μM celecoxib (Santa Cruz Biotechnology, Cat. No. sc-217869). The control cells were treated with vehicle (0.1% DMSO) only.

Treatment groups (*n* = 6 wells/group) were as follows: (i) NS; (ii) NS + PGE2; (iii) NS + celecoxib; (iv) HS; and (v) HS + celecoxib.

After 2 or 24 h, the media samples were collected for ELISA measurements. The remaining cells were trypsinized with 0.25% trypsin-EDTA (Gibco, Cat. No. T4049) and centrifuged, and pellets were collected for Real-time reverse transcription PCR (qRT-PCR) measurements. Results were obtained from single experiments using technical repeats. In the case of independent experiments, their number is indicated in the legend of the corresponding figure.

### Cell Proliferation and Cytotoxicity Assays

The effect of the applied Na^+^ concentrations and/or drug doses on cell proliferation and cytotoxicity were measured with MTT and LDH assays, respectively.

The cell viability of cultured human dermal fibroblast cells was detected by MTT assay. Cells were seeded in a 96-well plate, each well containing 5 × 10^3^ cells and treated with different NaCl concentrations (150, 175, 200, and 250 mM) for 24 h. MTT (5 mg/ml, Sigma-Aldrich, Shanghai, China) was added to each well, and the plate was incubated for another 4 h, then 150 μl DMSO (Sigma-Aldrich) was added. The plate was slowly oscillated until all the crystal substance was dissolved. Optical density (OD) was measured using a SpectroStar Nano microplate reader (BMG Labtech, Ortenberg, Germany) at 490 nm.

For LDH cytotoxicity assay, cultured human dermal fibroblast cells were seeded into 96-well plates at a density of 4 × 10^3^ cells/well (*n* = 6 well/treatment group) and treated with different NaCl concentrations (150, 175, 200, and 250 mmol/L) for 24 h. The LDH assay was performed as previously described (Korzeniewski and Callewaert, 1983). All reagents were purchased from Sigma-Aldrich. The absorbance was recorded at 570 and 690 nm as background in a SpectroStar Nano microplate reader (BMG Labtech, Ortenberg, Germany).

### Messenger RNA Isolation and cDNA Synthesis

Total RNA was isolated from skin samples or cell pellets using Total RNA Mini Kit (Geneaid Biotech, Cat. No. RT050, New Taipei City, Taiwan). The quality and quantity of the isolated RNA was determined with a NanoDrop ND-1000 spectrophotometer (Baylor College of Medicine, Houston, TX, United States). Thereafter, 500 ng total RNA was reverse-transcribed using Maxima First Strand cDNA Synthesis Kit for qRT-PCR (Thermo Fisher Scientific, Cat No. K1642) according to the manufacturer’s instructions.

### Real-Time Reverse Transcription Polymerase Chain Reaction

The mRNA expression of each sample was measured in triplicate with 20 μl/well final volume: 1 μl cDNA sample, 1 μl of 10 μM forward and reverse primers (IDT, Coralville, IA, United States), 7 μl PCR-grade water (Sigma-Aldrich, Cat. No. 3315932001), 10 μl 2× SensiFAST™ SYBR® No-ROX mix (Bioline, Cat. No. BIO-98005, London, UK). A LightCycler 480 thermocycler (Roche Diagnostics, Mannheim, Germany) was used to perform PCR. Cycle parameters included an initial denaturation step (95°C for 2 min), followed by a three-step cycling: denaturation (95°C for 5 s), annealing (55–62°C for 5 s), and extension (72°C for 5–8 s), for 40 cycles. Exact annealing temperatures and extension times can be found in Table 1 for each primer pair. The results were analyzed by LightCycler 480 software version 1.5.0 (Roche, RRID:SCR_012155) using the ΔΔCt method. Data were normalized against mRNA expression of 18S ribosomal RNA (RN18S, rat tissue) or glyceraldehyde-3-phosphate dehydrogenase (GAPDH, human dermal fibroblast cells) as an internal control and were presented as the ratio of their control values. Primers were designed by Lasergene PrimerSelect software version 7.1.0 (DNAStar, RRID:SCR_016295) based on nucleotide sequences from the National Center for Biotechnology Information’s nucleotide database (Table 1).

**Table 1:** Primer sequences and parameters.

Name	NCBI ID	Species	Primer sequences	T_a_ (°C)	Product length (bp)
rCOX2	NM_017232	Rat	F: 5'‐ GTTCGCATTCTTTGCCCAGC-3'	58	199
			R: 5'‐ AGGATACACCTCTCCACCGAT-3'		
rRN18S	NR_046237.1	Rat	F: 5'‐ GCGGTCGGCGTCCCCCAACTTCTT-3'	60	105
			R: 5'‐ GCGCGTGCAGCCCCGGACATCTA-3'		
hCOX1	NM_000962.3	Human	F: 5'‐ AGACGACCCGCCTCATCCTCATAG-3'	60	115
			R: 5'‐ CCGAACAGCAGCTCTGGGTCAAAT-3'		
hCOX2	NM_000963.3	Human	F: 5'‐ AATGGGGTGATGAGCAGTTGTTC-3'	58	202
			R: 5'‐ GGATGCCAGTGATAGAGGGTGTTA-3'		
hEP2	NM_000956.3	Human	F: 5'‐ CCATCACCTTCGCCGTCTGCT-3'	60	191
			R: 5'‐ CCGACAACAGAGGACTGAACGCATT-3'		
hEP4	NM_000958.2	Human	F: 5'‐ TCGTGGTGCGAGTATTCGTCAACC-3'	61	288
			R: 5'‐ CCCGGGAGATGAAGGAGCGAGAGT-3'		
hHAS1	NM_001523.3	Human	F: 5'‐ AATGTGGAGCGGGCTTGTCAGAG-3'	62	158
			R: 5'‐ AGGTGCCGGTCATCCCCAAAAGTA-3'		
hHAS2	NM_005328.2	Human	F: 5'‐ AGGGTCCCGGTGAGACAGATGAGT-3'	60	200
			R: 5'‐ TGAGGCTGGGTCAAGCATAGTGTC-3'		
hGAPDH	NM_002046.7	Human	F: 5'‐ AGCAATGCCTCCTGCACCACCAA-3'	60	159
			R: 5'‐ GCGGCCATCACGCCACAGTTT-3'		

Gene identification numbers – (NCBI ID), taken from the National Center for Biotechnology Information database – nucleotide sequences of forward (F) and reverse (R) primers, specific optimal annealing temperatures (T_a_) and product lengths are shown.

### Enzyme-Linked Immunosorbent Assay

The quantity of PGE2 and HA in the cell culture media was determined according to the manufacturer’s instructions by using both the High Sensitivity PGE2 ELISA Kit (Abcam, Cat. No. ab133055, Cambridge, UK) and the Hyaluronic Acid ELISA Kit (R&D Systems, Cat. No. HYAL0), respectively. Both the standards and samples were run in duplicates. Optical density (OD) was measured using a SpectroStar Nano microplate reader (BMG Labtech).

### Statistical Analyses

Data were analyzed using GraphPad Prism 7.0 Software (GraphPad Software, La Jolla, CA). Kolmogorov-Smirnov normality test was performed to determine the type of distribution of the values. In the case of parametric values, an unpaired Student’s *t*-test (two groups) or an ordinary one-way ANOVA followed by a Tukey’s *post-hoc* test (three or more groups) was applied. To analyze non-parametric data, a Mann-Whitney U rank test (two groups) or a Kruskal-Wallis rank test followed by a Dunn’s multiple-comparisons test (three or more groups) was performed. Data are presented as means ± SD (parametric) or medians ± interquartile range (non-parametric). A two-tailed value of *p* < 0.05 was considered to be statistically significant.

## Results

### Determination of Optimal Na^+^ Concentrations With *in vitro* Cell Viability Assays

**Figure 1:**
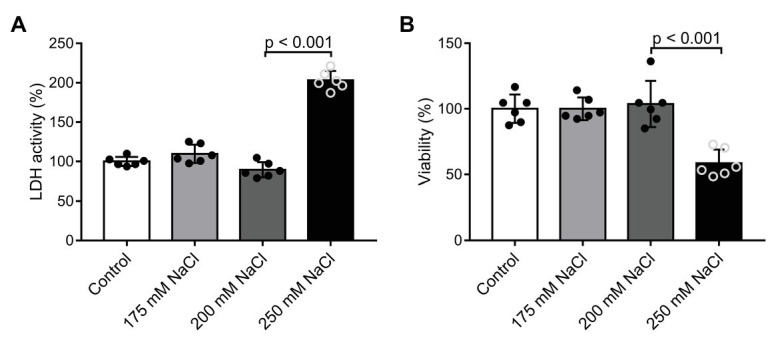
Determination of optimal sodium (Na^+^) concentrations with *in vitro* cell viability assays: **(A)** LDH cytotoxicity assay, and **(B)** MTT cell viability assay performed on cultured human dermal fibroblast cells after 24 h. Na^+^ concentration in the control media was 150 mM. *n* = 6 wells/group. One-way ANOVA test was performed. Data are presented as mean ± SD. Authors state that the number of independent experiments in reference to cell viability (MTT assay) was 3, and that the one shown is 1 representative of the corresponding repeats.

Na^+^ concentration 50 mmol/L higher than that of the control group (150 mmol/L) left both cytotoxicity (LDH assay) and cell viability (MTT assay) unchanged (Figures 1A,B). In contrast, Na^+^ at a concentration of 250 mmol/L resulted in cell damage.

### Examination of COX-2/PGE2 Pathway Activity in Salt Overloaded Human DFs

**Figure 2:**
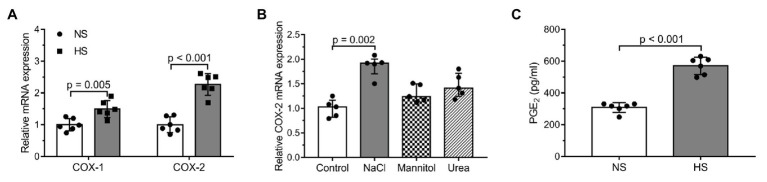
The examination of cyclooxygenase-2 (COX-2)/prostaglandin E2 (PGE2) pathway activity in salt overloaded human dermal fibroblasts after 24 h: **(A)** messenger RNA (mRNA) expression levels of the enzymes COX-1 and COX-2. **(B)** mRNA expression levels of the enzyme COX-2 in the media of cultured human dermal fibroblasts exposed to normal (150 mM NaCl = 300 mOsm/L) and high salt (200 mM NaCl = 400 mOsm/L) environment and the osmolytes mannitol (150 mM NaCl + 100 mM mannitol = 400 mOsm/L) and urea (150 mM NaCl + 100 mM urea = 400 mOsm/L). **(C)** PGE2 levels in the media of cultured human dermal fibroblasts. *n* = 5–6 wells/group. mRNA expression was normalized to GAPDH. NS, normal Na+ concentration and HS, high Na+ concentration. Unpaired t-test **(A,C)** and Kruskal-Wallis test **(B)** were performed. Data are presented as mean ± SD **(A,C)** or median ± interquartile range **(B)**. Authors state that the number of independent experiments in reference to COX-2 mRNA expression, as well as PGE2 concentration was 3 and 2 respectively, and that the one shown is 1 representative of the corresponding repeats.

In the cultured human DF cells, the mRNA expression levels of both COX-1 and COX-2 increased due to higher Na^+^ concentration; however, the rise in COX-2 expression was higher [COX-1: NS (normal sodium, 150 mmol/L): 0.9983 ± 0.1928 (arbitrary unit); HS (high sodium, 200 mmol/L): 1.493 ± 0.2711; COX-2: NS: 1.0 ± 0.2496; HS: 2.268 ± 0.3372; Figure 2A]. However, control osmolytes (mannitol or urea) failed to exert a similar effect (control: 1.030 ± 0.345, NaCl: 1.920 ± 0.305, mannitol: 1.240 ± 0.350, and urea: 1.410 ± 0.470; Figure 2B). In accordance with increased COX-2 mRNA expression, the concentration of PGE2 in the media increased, too (NS: 308 ± 31.16 pg/ml; HS: 570.9 ± 54.28 pg/ml; Figure 2C). The expression levels of the two receptors of PGE2 – PGE2 receptor (EP) 2 and 4 – were also measured: in the salt overloaded cells, the expression of EP2 decreased, whereas no change was detected in the expression of EP4 (EP2: NS: 1.0 ± 0.2286 arbitrary unit; HS: 0.3633 ± 0.1076; EP4: NS: 1.0 ± 0.0860; HS: 0.97 ± 0.2259; Supplementary Figure 1).

### Changes in the Expression Levels of HA Synthases and in the Production of HA in Salt Overloaded Human DFs

**Figure 3:**
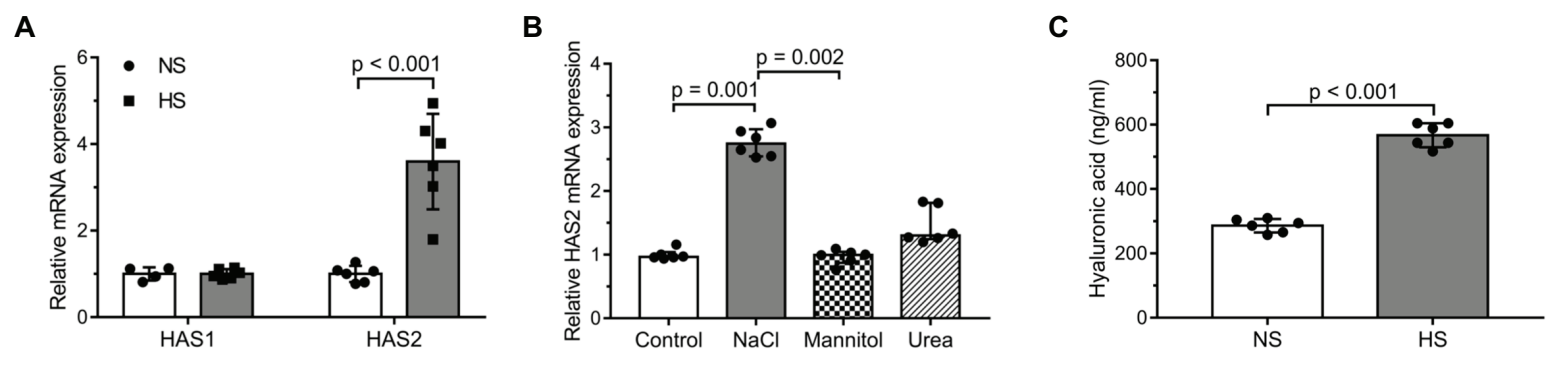
Changes in the expression levels of HA synthases (HAS) and in the production of HA in salt overloaded DFs after 24 h: **(A)** mRNA expression levels of the enzymes HAS1 and HAS2 in cultured human dermal fibroblasts in normal and high salt environment. **(B)** mRNA expression levels of the enzyme HAS2 in the media of cultured human dermal fibroblasts exposed to normal (150 mM NaCl = 300 mOsm/L) and high salt (200 mM NaCl = 400 mOsm/L) environment and the osmolytes mannitol (150 mM NaCl + 100 mM mannitol = 400 mOsm/L) and urea (150 mM NaCl + 100 mM urea = 400 mOsm/L). **(C)** Hyaluronic acid concentration measured in the media of cultured human dermal fibroblasts exposed to normal or high salt conditions. *n* = 6 wells/group. mRNA expression was normalized to GAPDH. NS, normal Na+ concentration and HS, high Na+ concentration. Unpaired t-test **(A,C)** and Kruskal-Wallis test **(B)** were performed. Data are presented as mean ± SD **(A,C)** or median ± interquartile range **(B)**. Authors state that the number of independent experiments in reference to HAS2 mRNA expression was 3, and that the one shown is 1 representative of the corresponding repeats.

From among the two isoforms of HAS, the mRNA expression of HAS2 exhibited change; it increased considerably following exposure to elevated levels of Na^+^ (HAS1: NS: 1.0 ± 0.1538 arbitrary unit; HS: 1.0 ± 0.1114; HAS2: NS: 0.9983 ± 0.1854; HS: 3.597 ± 1.1; Figure 3A). The rise was not induced by osmotic changes since only NaCl was effective in enhancing HAS2 mRNA, while the control osmolytes (mannitol or urea) failed to exert a similar effect (control: 0.965 ± 0.085, NaCl: 2.745 ± 0.428, mannitol: 0.995 ± 0.1725, and urea: 1.3 ± 0.057; Figure 3B). Measuring the amount of HA in the media, a significant elevation was found in the HS group (NS: 286.2 ± 20.94 ng/ml, HS: 567 ± 37.12 ng/ml; Figure 3C).

### Demonstration of the Relationship Between the COX-2 Pathway and HA Synthesis

**Figure 4:**
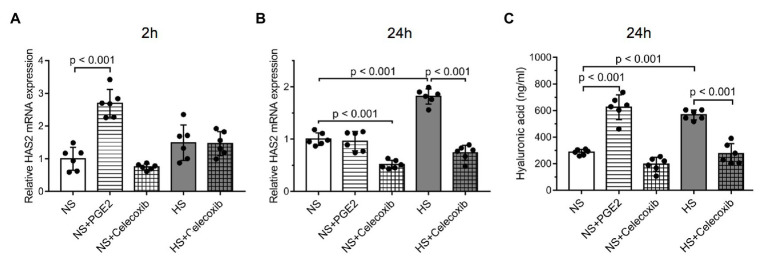
Interconnectivity between the COX-2 pathway and HA synthesis: The effect of five different treatment regimes on **(A)** the relative mRNA expression of the enzyme HAS2 measured at 2 h, **(B)** the relative mRNA expression of the enzyme HAS2 measured at 24 h, **(C)** the hyaluronic acid concentrations measured in the media of cultured human dermal fibroblasts. *n* = 6 wells/group. mRNA expression was normalized to glyceraldehyde-3-phosphate dehydrogenase (GAPDH). NS, normal Na+ concentration and HS, high Na+ concentration. One-way ANOVA tests were performed. Data are presented as mean ± SD. Authors state that results were obtained from single experiments using technical repeats.

PGE2 administration alone resulted in higher mRNA expression of HAS2 at 2 h (NS: 0.9983 ± 0.3514, NS + PGE2: 2.695 ± 0.4282, NS + Celecoxib: 0.75 ± 0.0918, HS: 1.49 ± 0.5443, HS + Celecoxib: 1.465 ± 0.3653; Figure 4A). After 24 h, the higher Na^+^ concentration achieved significantly higher mRNA expression (NS: 1.002 ± 0.1189, NS + PGE2: 0.9617 ± 0.1843, NS + Celecoxib: 0.5133 ± 0.08042, HS: 1.815 ± 0.1469, HS + Celecoxib: 0.7383 ± 0.1459; Figure 4B). The effect of celecoxib was significant after 24 h in the NS + celecoxib group, it decreased gene expression to a level lower than that of the NS group (Figure 4B). When administered in combination with a high Na^+^ level, it blocked the HAS2 increase that develops in the cells that were treated with Na^+^ alone (Figure 4B).

The amount of the end product HA in the media was increased after 24 h as a result of PGE2 and a higher Na^+^ concentration, too (NS: 286.2 ± 20.94 ng/ml, NS + PGE2: 624.6 ± 92.75 ng/ml, NS + Celecoxib: 195.1 ± 53.31 ng/ml, HS: 567 ± 37.12 ng/ml, HS + Celecoxib: 274 ± 6.54 ng/ml; Figure 4C). The effect of the latter was blocked with celecoxib.

### The Combined Effect of Salt Overload and Celecoxib Administration on Na^+^ Concentration of Serum and on Urinary Na^+^ Excretion

The level of serum Na^+^ was slightly higher in group HSD + V, although in all three groups it remained within the physiological range [LSD + V (low-salt diet + vehicle): 139.0 ± 3.0 mmol/L, HSD + V (high-salt diet + vehicle): 141.5 ± 4.0 mmol/L, HSD + C (high-salt diet + celecoxib): 140.0 ± 1.0 mmol/L; Supplementary Figure 3]. Urinary Na^+^ excretion – expressed as the ratio of urinary Na^+^ and creatinine concentration – in group HSD + V was higher than in group LSD + V, as it was predictable from the elevated dietary Na^+^ intake (LSD + V: 22.3 ± 10.4, HSD + V: 389.7 ± 176.2; Supplementary Figure 4). In group HSD + C, no significant decrease in urinary Na^+^ excretion was found (HSD + C: 232.4 ± 142.4).

### The Combined Effect of Salt Overload and Celecoxib Administration on BP

Salt overload alone did not increase the systolic BP (LSD + V: 117.7 ± 11.9 mmHg, HSD + V: 127.4 ± 10.4 mmHg; Supplementary Figure 5). However, when combined with the administration of celecoxib, the systolic BP rose significantly (HSD + C: 137.4 ± 8.6 mmHg).

### The Combined Effect of Salt Overload and Celecoxib Administration on the mRNA Expression of COX-2 as Well as on the Content of Na^+^, CS‐ and HA-Disaccharides in the Skin

**Figure 5:**
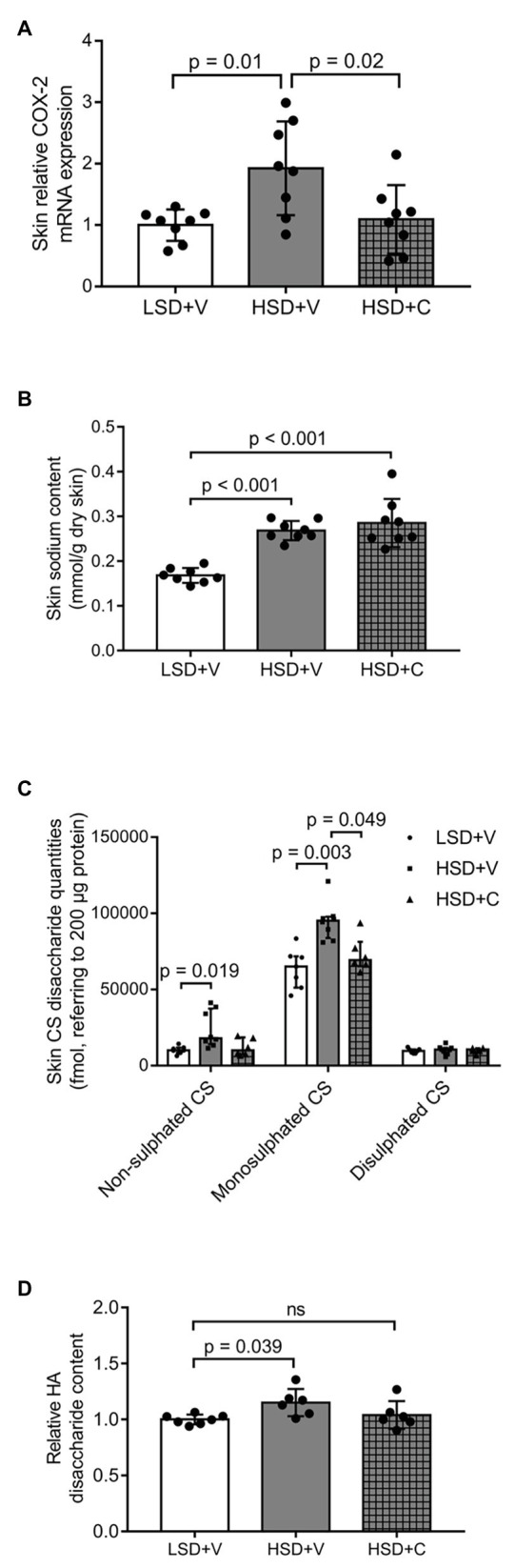
Effect of salt overload and celecoxib administration on the content of Na^+^, chondroitin-sulfate, and hyaluronic acid disaccharides, as well as on the mRNA expression of COX-2 in the skin: **(A)** mRNA expression levels of the enzyme COX-2 in the skin of rats. Contents of **(B)** Na^+^, **(C)** chondroitin-sulfate, and **(D)** hyaluronic acid disaccharides of rat skin. The chondroitin-sulfate disaccharide content of the skin refers to 200 μg protein (non-sulfated, monosulfated, and disulfated disaccharides). *n* = 6–8 rats/group. The mRNA expression was normalized to 18S RNA. LSD + V: low-salt diet + vehicle; HSD + V: high salt-diet + vehicle; HSD + C: high salt diet + celecoxib; CS, chondroitin-sulfate; HA, hyaluronic acid; and ns, not significant. Kruskal-Wallis test **(A)** and one-way ANOVA tests **(B–D)** were performed. Data are presented as median ± interquartile range **(A)** or mean ± SD **(B–D)**. Authors state that results were obtained from a single experiment.

Following salt overload, the COX-2 mRNA expression increased in the skin [LSD + V: 1.0 ± 0.2543 (arbitrary unit), HSD + V: 1.926 ± 0.7635; Figure 5A]. Additional administration of celecoxib prevented this elevation (HSD + C: 1.094 ± 0.5576; Figure 5A). The Na^+^ content of the skin in salt overloaded groups increased compared to the one in the LSD + V group; however, no difference between the Na^+^ content of HSD + V and HSD + C groups was measured (LSD + V: 0.168 ± 0.017 mmol/g dry skin, HSD + V: 0.268 ± 0.021 mmol/g dry skin, HSD + C: 0.285 ± 0.054 mmol/g dry skin; Figure 5B). Furthermore, the content of CS in the skin was affected by the applied treatments, as well: the amount of both non-sulfated and monosulfated CS disaccharides significantly increased in the HSD + V group compared to the one in the LSD + V group (non-sulfated CS: LSD + V: 10,292 ± 2,489 fmol, HSD + V: 23,861 ± 12,089 fmol; nonosulfated CS: LSD + V: 63,763 ± 13,021 fmol, HSD + V: 94,835 ± 12,480 fmol; Figure 5C). Interestingly, inhibition of COX-2 blocked the overproduction of monosulfated CS disaccharides in the skin of salt overloaded rats (HSD + C: 72,942 ± 11,422 fmol; Figure 5C). In the case of disulfated CS, no difference was observed among groups (Figure 5C). Finally, the relative HA disaccharide content was increased in the skin of HSD + V rats compared to that of LSD + V animals (LSD + V: 1.000 ± 0.043, HSD + V: 1.151 ± 0.122); however, no elevation was observed in the HSD + C group (HSD + C: 1.040 ± 1.124; Figure 5D).

## Discussion

We demonstrated with our results for the first time that under salt overloaded condition GAG production in the skin is regulated by the local prostaglandin system. The elevated extracellular Na^+^ concentration activates the COX-2/PGE2 pathway in human dermal fibroblasts, which results in increased HA production. The relationship between the two regulatory systems was demonstrated by using cell culture and animal model, as well.

Our results demonstrate that dermal fibroblasts retain their viability in a cell culture if the Na^+^ concentration is not exceeding 200 mmol/L (Figures 1A,B). This is in line with *in vitro* salt overload experiments and consistent with experimental interstitial tissue Na^+^ concentrations (180–200 mmol/L) measured *in vivo* following dietary salt load (Müller et al., 2013; Wiig et al., 2013; Zhang et al., 2015).

The *in vitro* measurements performed on dermal fibroblasts proved that not only macrophages and keratinocytes, but dermal fibroblasts are also involved in the regulation and storage of skin Na^+^ content (Xu et al., 2015; Zhang et al., 2015). One possible pathway is the COX-2/PGE2 pathway, which is expressed in both dermal macrophages and keratinocytes and responds to various stimuli, i.e., higher skin Na^+^ concentration. We demonstrated a Na^+^ induced activation of the COX/PGE2 pathway in DF cells. In cultured human DF cells, mRNA expression levels of both COX-1 and COX-2 increased due to higher Na^+^ concentration (Figure 2A). This increase can be observed even in the constitutively expressed COX-1 isoform; however, to a lesser extent than in COX-2. Since control osmolytes did not exert similar effect, our data proved the specific, osmolarity independent effect of NaCl on COX-2 mRNA expression (Figure 2B). In accordance with the increased COX-2 mRNA expression, elevated concentration of PGE2 in the media was found (Figure 2C). Regarding the PGE2 receptors, the expression levels of the two receptors of PGE2 (EP2 and EP4) showed different response in salt overloaded cells; the mRNA expression of EP2 decreased, whereas no change was detected in the expression of EP4 (Supplementary Figure 1). A possible explanation for the downregulation of EP2 receptor is receptor desensitization, which is an indirect evidence of PGE2 rise. Interestingly, we found instances in the literature which contradict the above pattern of EP receptor mRNA expression; PGE2 caused the downregulation of EP4 and the upregulation of EP2 receptor in macrophages and fibroblasts (Ikegami et al., 2001; Shim, 2019). Therefore, the gene expressions of EP2 and EP4 are differentially regulated, the direction of change in gene expression probably depends on the trigger.

In a salt-loaded condition, the organism tries to bind and store the unnecessary Na^+^ mainly in the skin. GAG molecules due to their high charge density are capable of sequestering positively charged ions and molecules (Farber et al., 1957). Therefore, extracellular GAGs are considered to play a crucial role in Na^+^ storage in salt loaded condition (Titze et al., 2004). There are two types of GAGs: the unsulfated HA and the sulfated GAGs. In the branched structure of GAGs, HA serves as a backbone for sulfated molecules. Since HA is an integrated part of all kinds of GAGs, our *in vitro* experiments focused on this macromolecule.

Specific enzymes, the HASs are responsible for the synthesis of HA; in mammals three, plasma membrane integrated isoforms are known. Among the isoforms, HAS1 and HAS2 synthetize longer chains of HA. In our cell culture experiments, only HAS2 was reactive to Na^+^ excess, and the increased HAS2 mRNA expression facilitated the overproduction of HA (Figure 3A). Such reaction of fibroblasts seems to be a compensatory and cell/tissue protecting cellular mechanism, which aims at neutralizing Na^+^. The use of osmotic controls (i.e., urea and mannitol) revealed that HAS2 expression is not solely a response to an increased external osmolality, but a specific result exerted by the NaCl solution independently of its osmotic properties (Figure 3B).

Since the Na^+^ load increased both HAS2 and COX-2 expressions of DFs, we investigated the possible relationship between these two systems. Administration of both PGE2 or Na^+^ load had an impact on HAS2 enzyme expression, although they exerted their effect at different time points (Figures 4A,B). This implies differences in the kinetics of the two signal transduction pathways. The fact that PGE2 was able to induce HA synthesis directly proves that PGE2 is a potent activator of HA synthesis. Moreover, the blocked overexpression of HAS2 with the impaired overproduction of HA 24 h after COX-2 inhibition in the media with high Na^+^ concentration indicates the interconnectivity of COX-2/PGE2 pathway and HA synthesis in a high Na^+^ environment (Figures 4B,C).

Data in the literature obtained from animal experiments proposed the involvement of COX-2/PGE2 pathway in the regulation of skin Na^+^ content. Higher Na^+^ concentration measured in the skin is likely to increase the COX-2 activity of macrophages and keratinocytes (Xu et al., 2015; Zhang et al., 2015). Our cell culture experiments supported the fact that COX-2 activation occurred in dermal fibroblasts too. Nevertheless, certain products of the enzyme COX-2 increase the HA production of DFs. On the one hand, DFs act in an autocrine/paracrine manner, in a way that one cell activates itself or its neighbors. On the other hand, COX-derivatives produced by nearby macrophages or keratinocytes may also trigger the DFs’ HA production.

In the animal experiment, our data confirm previous observations, where the overall expression of COX-2 mRNA increased in response to salt overload (Figure 5A; Zhang et al., 2015). Elevated COX-2 mRNA expression is evidently an integrated effect of different cell types. When COX-2 was inhibited in the salt loaded animals (HSD + C) in our experiments, the COX-2 mRNA expression exhibited a similar level of the control group. This fact certainly proves the pharmacological blockade of the whole system, which is a phenomenon attributed to the cessation of COX-2’s positive feedback effect.

No biologically relevant change in the level of serum Na^+^ was detected; serum Na^+^ concentration remained in the physiological range even in the salt overloaded groups (Supplementary Figure 3). In contrast to serum Na^+^ content, skin Na^+^ levels exhibited significant change, which is in line with literature data (Titze et al., 2003; Wiig et al., 2013; Figure 5B). Surprisingly, no reduction in Na^+^ content was detected when high Na^+^ diet, and COX-2 inhibition was administered simultaneously. We hypothesized that skin Na^+^ content reduces in line with GAG synthesis. After all, Na^+^ content was not reduced by COX-2 inhibition. The answer probably lies in the complex effect of COX-2 inhibition on Na^+^ homeostasis. COX-2 inhibition may have increased skin Na^+^ content by affecting two distinct processes: either by inhibiting lymph capillary proliferation of the skin and disturbing the clearance of tissue Na^+^, or by facilitating the expression of Na^+^ channels in the kidney and consequent Na^+^ reabsorption. Although we did not find any significant decrease in urinary Na^+^ excretion in the case of COX-2 inhibition (Supplementary Figure 4), it is a well-known effect in the literature (Harirforoosh and Jamali, 2005; Yang and Liu, 2017).

According to literature data, high salt consumption increases the amount of GAGs of the skin. Our findings corroborate this phenomenon; the amount of both CS‐ (non-sulfated and monosulfated) and HA-disaccharides increased in the skin of salt overloaded experimental animals (Figures 5C,D). Concomitant COX-2 inhibition blocked the overproduction of both GAGs (Figures 5C,D), suggesting that COX-2 is involved not only in the regulation of HA but also in sulfated GAGs. The results from skin HA measurement are in line with the data regarding HA content in cell cultures and CS content in the skin during salt overload. Therefore, these results support the proposed mechanism of the effect of COX-2 inhibition on the regulation of GAGs.

**Figure 6:**
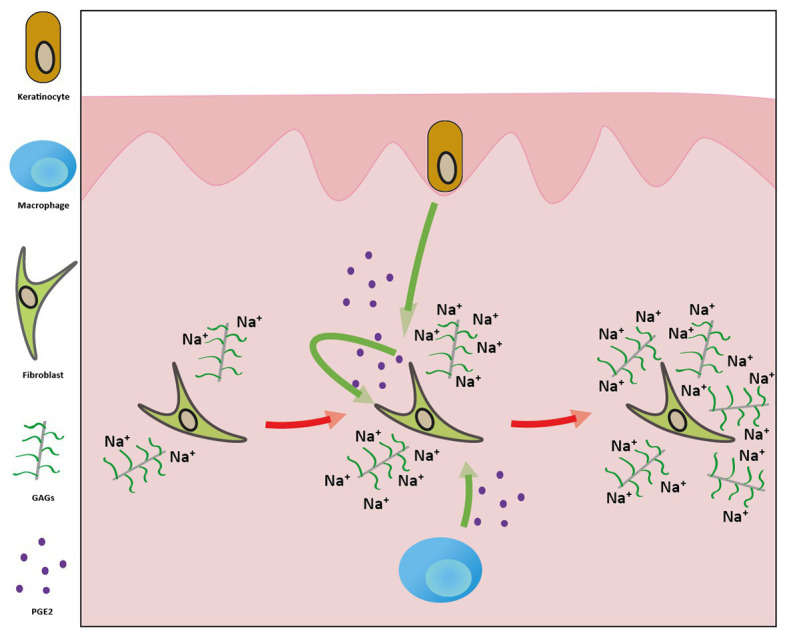
The mechanism by which the high-sodium environment promotes increased glycosaminoglycan (GAG) production in the skin. The elevated extracellular Na^+^ concentration activates the COX-2/PGE2 pathway in keratinocytes, macrophages, and dermal fibroblasts. The resulting PGE2 molecules exert their effect in both autocrine and paracrine manners on dermal fibroblasts, leading to increased synthesis of GAGs. These negatively charged macromolecules can bind more sodium ions in the interstitium.

These findings are in contrast with the observation of Zhang et al. (2015), who described the opposite effect; the Na^+^ content of the skin grew even further in salt overloaded animals. In their experiment, only the enzyme of skin-infiltrating macrophages had been knocked out, keratinocytes and DFs retained their COX-2 activity; therefore, presumably they also retained their ability to produce GAGs. In our experiment, COX-2 inhibition was achieved by *Celecoxib* administration: a treatment affecting enzyme activity in all cells of the body. Methodical differences between the two studies may give explanation to the contradictory results; in the article of Zhang et al. (2015), COX-2 inhibition increased skin Na^+^ content, whereas in our experiment similar intervention left skin Na^+^ level unchanged. In our case, COX-2 activity of macrophages, keratinocytes, and fibroblasts in the skin decreased, which – in light of our results – could have repressed GAG production in the skin. Thus, Na^+^ accumulated in the body – resulting from either inhibition of lymph capillary proliferation or increased Na^+^ retention in the kidneys – was probably unable to bind to GAGs and increase Na^+^ content of the skin.

No change in BP was measured in the animals of group HSD + V – in line with the fact that the Wistar strain used is considered to be a relatively salt resistant one (Supplementary Figure 5). In contrast, elevated BP developed in the group receiving the combination of high Na^+^ and celecoxib treatment (HSD + C). Three mechanisms may explain the hypertensive effect of the drug: (1) Na^+^ retention in the kidney, (2) cessation of the vasodilatory effect resulting from decreased prostacyclin production of the blood vessel wall, and (3) reduced lymph vessel formation (Harirforoosh and Jamali, 2005; Yu et al., 2010; Zhang et al., 2015; Yang and Liu, 2017). Apart from these documented effects of COX-2 inhibition, the decreased production of GAGs (i.e., deteriorated Na^+^-binding capacity of the skin) may have also contributed to the increased BP.

However, the lack of a control group on a standard-salt diet is a possible limitation of our experiment. The experimental design used in our study (low-salt diet vs. high-salt diet) is a well-established model in the literature, despite exposing both groups to the extremes of sodium ingestion during the course of the experiment (Titze et al., 2002, 2003, 2004; Machnik et al., 2009; Jantsch et al., 2015; Nikpey et al., 2017; Benz et al., 2018). In this model, instead of using a standard-salt diet group, the high-salt diet induced changes are compared directly to the state of salt deprivation. In the literature, the use of these two dietary groups resulted in clear difference in skin sodium content, and certain pathophysiological alterations were associated with this difference. In our own experiments, too, we attributed pathophysiological changes to differences in skin salt content. Moreover, these findings were strongly supported by our *in vitro* experiments, too. However, using an additional normal-salt diet group, in which the consumed amount of sodium would meet the animals’ needs, it would provide a more detailed picture of the relationship of salt overload and COX-2-mediated GAG production in the skin.

In summary, we demonstrated that high-sodium environment increases GAG synthesis of dermal fibroblasts *via* the activation of the COX-2/PGE2 pathway (Figure 6). Beside other cell types, dermal fibroblasts are also capable of producing PGE2. The resulting PGE2 molecules act on fibroblasts in both paracrine and autocrine manners. Moreover, the salt overloaded Wistar rats treated with the COX-2 inhibitor celecoxib failed to increase the amount of Na^+^-binding GAGs in the skin. Taken together, in case of salt overload, GAG production in the skin is regulated by the local prostaglandin system. These data provide further insight into the fibroblast-mediated regulation of GAGs in the skin during high salt consumption, as well as into the dermal Na^+^ homeostasis.

## Data Availability Statement

The raw data supporting the conclusions of this article will be made available by the authors, without undue reservation.

## Ethics Statement

The animal study was reviewed and approved by Committee of the Care and Use of Laboratory Animals in the Council of Animal Care at Semmelweis University, Budapest, Hungary (PEI/001/1731-9/2015).

## Author Contributions

RA and AS conceived and designed the research. RA, DS, and DP performed animal and cell culture experiments. GT and LT designed and performed HPLC-MS measurements. LK and ZV gave scientific advice in the field of dermatology. RA and DP analyzed data. RA, DP, TT, ÁV, and AS interpreted results of experiments. RA prepared figures. RA and DS drafted manuscript. DP, TT, ÁV, and AS edited and revised the manuscript. RA, DS, DP, TT, ÁV, AS, GT, LT, and LK approved final version of the manuscript. All authors contributed to the article and approved the submitted version.

### Conflict of Interest

The authors declare that the research was conducted in the absence of any commercial or financial relationships that could be construed as a potential conflict of interest.

## Abbreviations

BP: Blood pressure
COX: Cyclooxygenase
CS: Chondroitin sulfate
DF: Dermal fibroblast
EP: Prostaglandin E2 receptor
GAG: Glycosaminoglycan
HA: Hyaluronic acid
HAS: Hyaluronic acid synthase
HSD: High-salt diet
LSD: Low-salt diet
Na^+^: Sodium
PGE2: Prostaglandin E2.
