# Supplementary material for: Cyclooxygenase-2 Modulates Glycosaminoglycan Production in the Skin During Salt Overload
Source: Front Physiol. 2020 Oct 23;11:561722. doi: 10.3389/fphys.2020.561722 (PMC7645107; doi:10.3389/fphys.2020.561722)
Supplement: Supplementary file 1 [file Data_Sheet_1.docx]

Supplementary Material

# Supplementary Figures and Tables


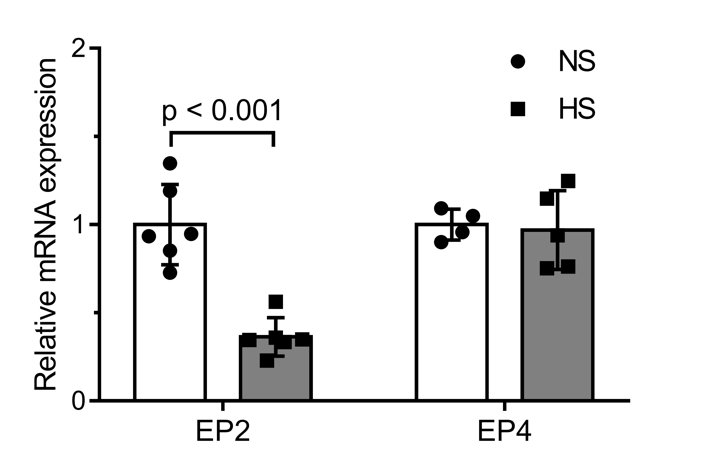


Supplementary Figure 1. Relative mRNA expression of the PGE2 receptors EP2 and EP4 in cultured human dermal fibroblasts in normal and high salt environment after 24 hours. n = 6 wells/group. mRNA expression was normalized to GAPDH. NS: normal salt, HS: high salt. Unpaired t-test was performed. Data are presented as Mean ± SD.

Supplementary Table 1. Data regarding body weight gain, caloric intake, skin sodium-, potassium- and water content, as well as diastolic blood pressure. n = 6-8 rats/group. LSD+V: low-salt diet + vehicle; HSD+V: high salt-diet + vehicle; HSD+C: high salt diet + Celecoxib. One-way ANOVA tests and Kruskal-Wallis tests (caloric intake and skin Na^+^+K^+^/water ratio) were performed. Data are presented as Mean ± SD or Median ± Interquartile range (caloric intake and skin Na^+^+K^+^/water ratio). *p < 0.05 vs. LSD+V group. #p < 0.01 vs. LSD+V group.

|  | LSD+V | HSD+V | HSD+C |
| --- | --- | --- | --- |
| Body weight  (regarding to initial weight, %) |  |  |  |
| Body weight gain  (Day 3 of treatment) | 115.5 ± 3.9 | 111.2 ± 4.5 | 108.4 ± 3.5 |
| Body weight gain  (Day 7 of treatment) | 120.4 ± 5.0 | 110.1 ± 3.9 # | 114.3 ± 4.3 * |
| Body weight gain  (Day 14 of treatment) | 127.2 ± 5.2 | 108.8 ± 3.3 # | 112.6 ± 6.1 # |
|  |  |  |  |
| Caloric intake / 2 weeks  of treatment (kcal) | 1295 ± 85 | 1312 ± 111 | 1201 ± 184 |
|  |  |  |  |
| Skin Na^+^ (mmol/g dry skin) | 0.168 ± 0.017 | 0.268 ± 0.021 # | 0.285 ± 0.054 # |
| Skin K^+^ (mmol/g dry skin) | 0.104 ± 0.070 | 0.116 ± 0.010 | 0.112 ± 0.012 |
| Skin water (ml/g dry skin) | 1.748 ± 0.443 | 2.302 ± 0.216 # | 2.247 ± 0.110 # |
| Skin (Na^+^ + K^+^) / skin water  (mmol/ml) | 0.174 ± 0.056 | 0.167 ± 0.012 | 0.169 ± 0.024 |
|  | | | |
| Diastolic blood pressure  (mmHg) | 80.4 ± 9.2 | 85.8 ± 7.2 | 95.2 ± 8.7 # |

**
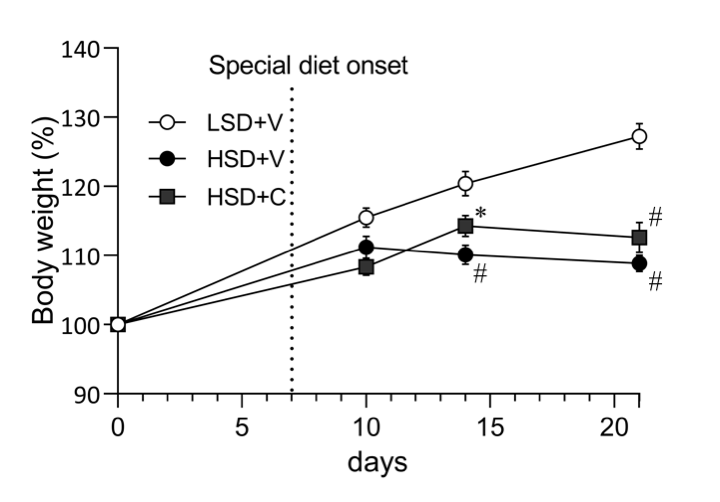
**

Supplementary Figure 2. Body weight gain during the experimental period. n = 8 rats/group. LSD+V: low-salt diet + vehicle; HSD+V: high salt-diet + vehicle; HSD+C: high salt diet + Celecoxib. Two-way ANOVA test was performed. Data are presented as Mean ± SEM. *p < 0.05 vs. LSD+V group. #p < 0.001 vs. LSD+V group.


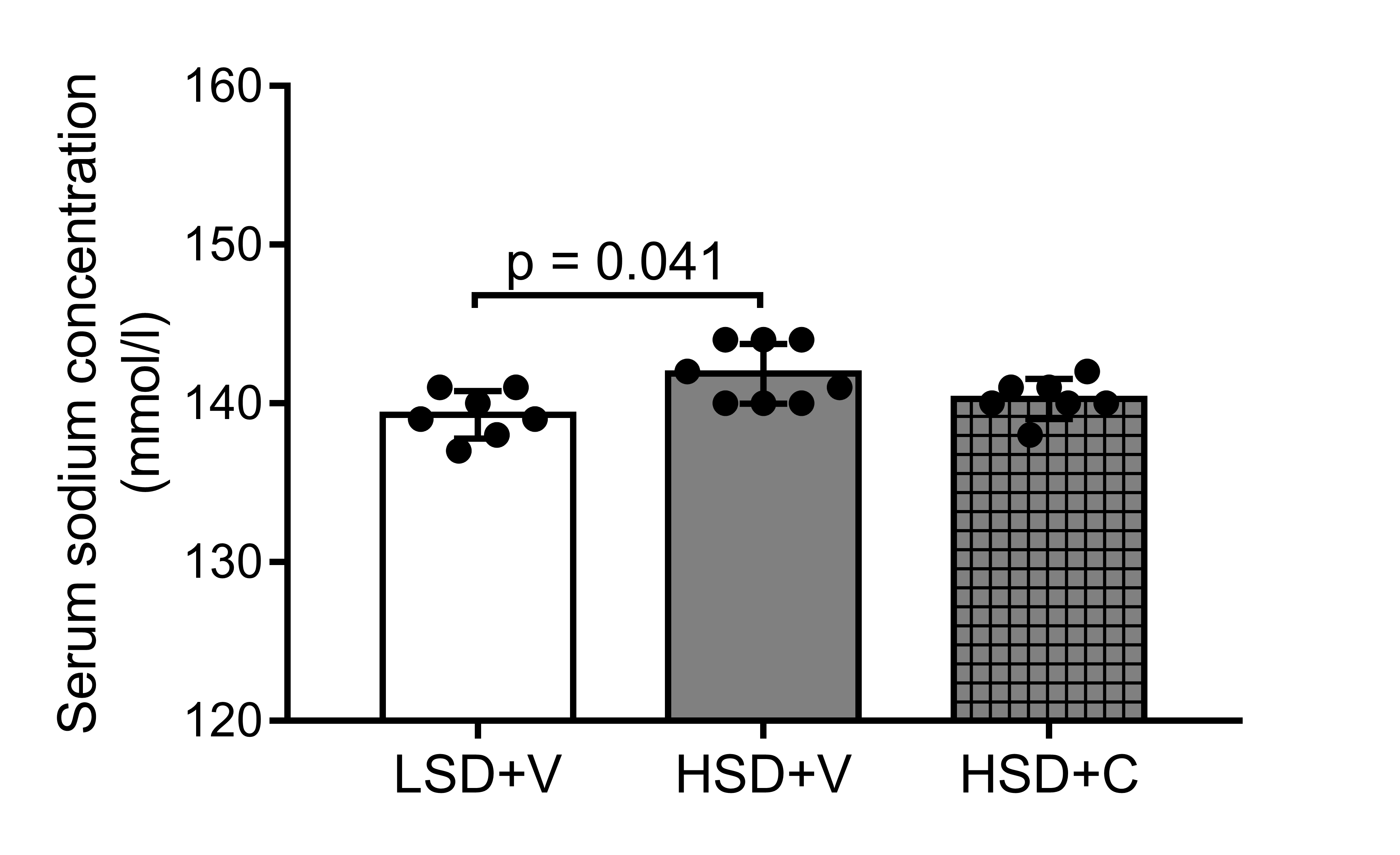


Supplementary Figure 3. Effect of salt overload and Celecoxib administration on Na+ concentration of serum: n = 7-8 rats/group. LSD+V: low-salt diet + vehicle; HSD+V: high salt-diet + vehicle; HSD+C: high salt diet + Celecoxib. Kruskal-Wallis test was performed. Data are presented as Median ± Interquartile range.


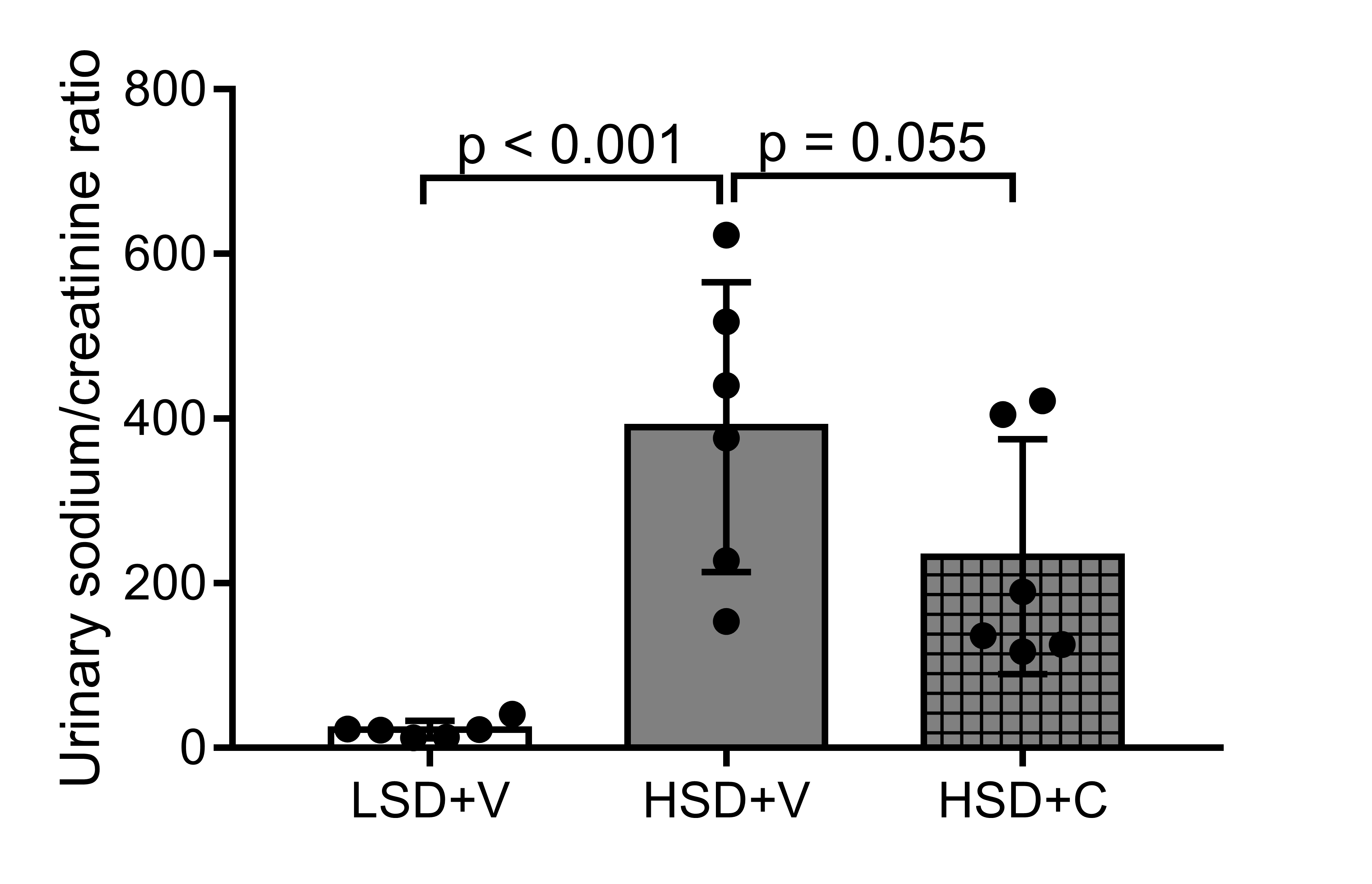


## Supplementary Figure 4. Effect of salt overload and Celecoxib administration on urinary Na^+^ excretion. n = 6 rats/group. LSD+V: low-salt diet + vehicle; HSD+V: high salt-diet + vehicle; HSD+C: high salt diet + Celecoxib. One-way ANOVA test was performed. Data are presented as Mean ± SD.


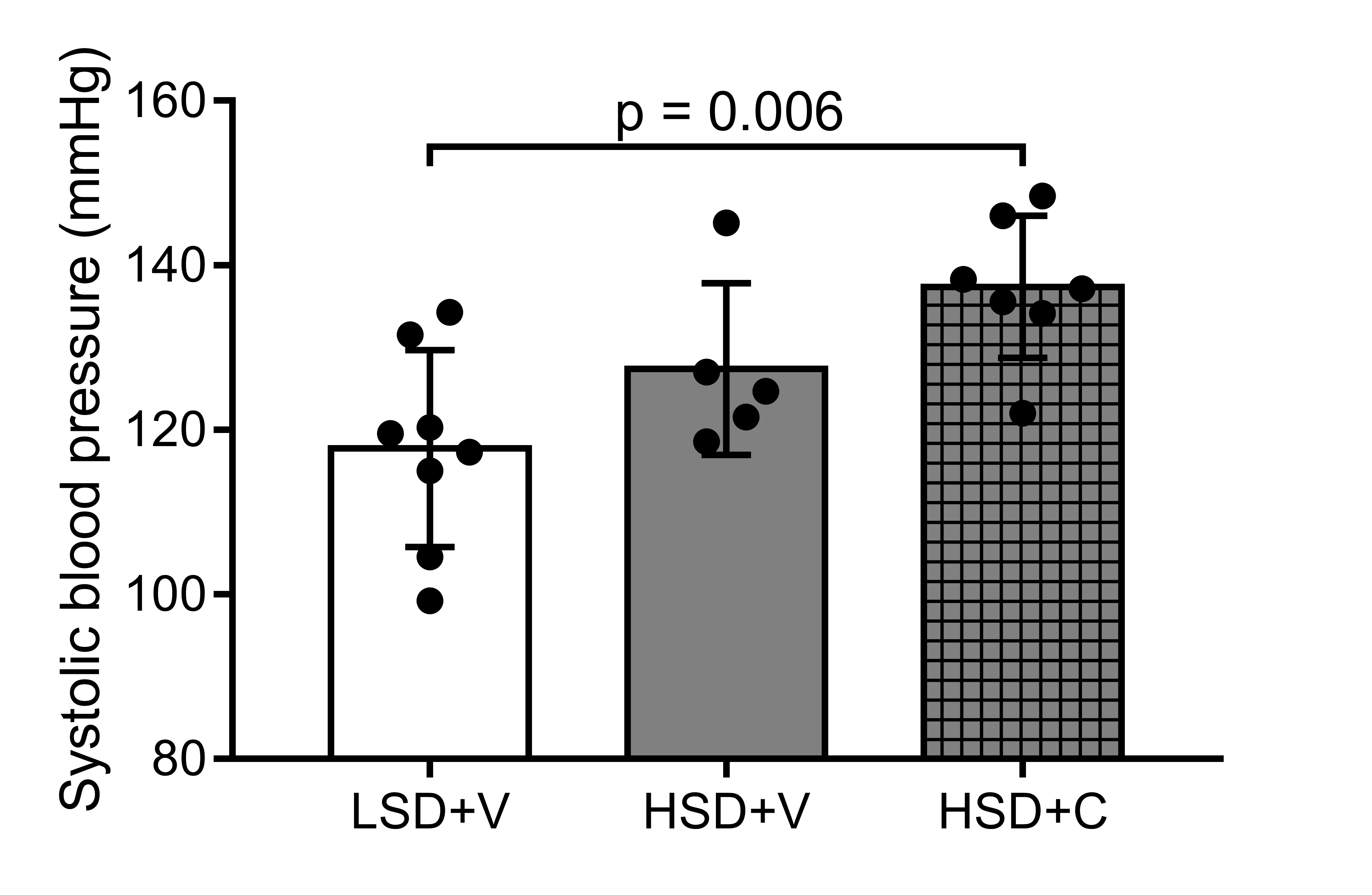


## Supplementary Figure 5. Effect of salt overload and Celecoxib administration on systolic blood pressure. n = 5-8 rats/group. LSD+V: low-salt diet + vehicle; HSD+V: high salt-diet + vehicle; HSD+C: high salt diet + Celecoxib. One-way ANOVA test was performed. Data are presented as Mean ± SD.


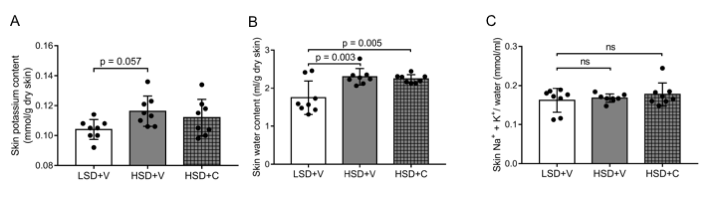


Supplementary Figure 6. Effect of salt overload and Celecoxib administration on the content (A) of K+ (B) and water, as well as (C) on the Na^+^ + K^+^/water ratio in rat skin. n = 8 rats/group. LSD+V: low-salt diet + vehicle; HSD+V: high salt-diet + vehicle; HSD+C: high salt diet + Celecoxib. One-way ANOVA tests (A and B) and Kruskal-Wallis test (C) were performed. Data are presented as Mean ± SD (A and B) or Median ± Interquartile range (C).
